# Editorial: The role of auxin in plant-microbe interactions

**DOI:** 10.3389/fpls.2024.1500649

**Published:** 2024-11-05

**Authors:** Barbara N. Kunkel, Jutta Ludwig-Müller, Ka-Wai Ma

**Affiliations:** ^1^ Department of Biology, Washington University in St. Louis, St. Louis, MO, United States; ^2^ Faculty of Biology, Technische Universität Dresden, Dresden, Germany; ^3^ Institute of Plant and Microbial Biology, Academia Sinica, Taipei, Taiwan

**Keywords:** auxin, bacteria, fungi, indole-3-acetic acid (IAA), microbiome, plant-microbe interactions, pathogens, symbionts

The plant hormone auxin regulates many important aspects of plant growth and development (Lavy and Estelle, 2016). There is growing evidence that auxin modulates the interactions between plant hosts and their associated microbes, including beneficial symbionts, endophytes and pathogenic organisms that cause disease. Thus, it comes as no surprise that auxin is also produced or catabolized by microbes to influence host signaling, physiology and development. Further, recent studies have revealed that auxin (especially indole-3-acetic acid, IAA) can act as a signaling molecule that directly impacts microbial development and/or gene expression (Kunkel and Johnson, 2021).

In this Research Topic, we invited researchers to submit articles investigating the various ways in which auxin influences host and/or microbial biology. Since putting out the call in late 2021, we received only a handful of manuscripts related to this topic. In hindsight, this is not unexpected, given how new this area of investigation is.

The four articles in this Research Topic report advances in synthesis and modification of IAA by plant-associated microbes and the roles of auxin in modulating plant-microbe interactions.

## Synthesis and modification of IAA by microbes

There is growing evidence that many Plant Growth Promoting Fungi (PGPF) can produce IAA. Chen et al. demonstrated in co-cultivation experiments with *Arabidopsis thaliana* that the ability of the PGPF *Penicillium citrinum* isolate B9 to stimulate plant growth appears to be due, at least in part, to production of IAA by the fungus. Their findings lead to several intriguing questions: 1) Does *P. citrinum* B9 produce IAA when growing in the natural rhizosphere community, in the presence of relevant host plants? and if so, 2) How does this IAA impact the growth of the plants? In the future, reverse genetics can be used to explore the roles of IAA produced by *P. citrinum* in the field, provided that researchers can identify the IAA synthesis genes and use genome editing to disrupt IAA synthesis in the fungus.

Some bacteria, such as the gall-forming woody plant pathogen *Pseudomonas savastanoi*, are not only able to synthesize IAA, but also a modified IAA amino acid conjugated form, IAA-Lys. IAA-Lys is believed to be less active than free IAA (Glass and Kosuge, 1988). Pintado et al. made the discovery that different strains of *P. savastanoi* carry different alleles of *iaaL*, the gene encoding the conjugating enzyme. Overexpression of different *iaaL* allozymes in *P. savastanoi* resulted in reduced free IAA levels and alterations in Arabidopsis root architecture in the presence of these strains. What is the biological relevance of these *iaaL* alleles? Since *P. savastanoi* is not a root pathogen, it is not clear how direct manipulation of root architecture would be beneficial to the bacterium. Alternatively, *iaaL* diversity may reflect selection for the ability of *P. savastanoi* to fine-tune free IAA levels in different hosts or infected tissues at various stages in pathogenesis. However, an impact of these *iaaL* alleles on pathogenesis was not observed in this study.

## Impact of auxin on plant development and modulation of plant-microbe interactions

The previous two studies provide examples of how microbe-derived auxins and auxin derivatives can modulate plant architecture. This property is potentially useful to improve plant traits with practical implications. In another study of the Research Topic, Chen et al. investigated the potential of an arbuscular mycorrhizal fungus (AMF) *Rhizophagus intraradices* to induce adventitious root formation in tea plant cuttings. Application of the auxin indole-3-butyric acid (IBA), had a marginal effect on increasing AMF colonization, possibly due to the ability of *R. intraradices* to produce auxin. The role of auxins in adventitious root formation and plant microbe interactions was further confirmed by the use of three auxin inhibitors that interfere with different steps of auxin biology, including biosynthesis, transport and perception. Their co-treatment with *R. intraradices* abolished the formation of adventitious roots in tea tree cuttings and reduced the root colonization on another host. Consistently, transcriptomic analysis suggested that *R. intraradices* altered the expression of many auxin-related genes in the host.

In addition to IAA, other molecules with slightly different structures behave as auxins. For example, the auxin phenylacetic acid (PAA) is found in several plant species and its biosynthesis follows the general IAA synthesis pathway in plants (Cook and Ross, 2016). This observation raises the possibility that plant-associated bacteria may also synthesize PAA (Zerrouk et al., 2020). As reported by Lee et al., the phytopathogenic bacteria *P. syringae Pto*DC3000 converts PAAld to PAA by using the AldA indole-3-acetaldehyde dehydrogenase enzyme, previously shown to convert IAAld to IAA. However, the ability of *Pto*DC3000 to synthesize either PAA or IAA does not appear to be important for the virulence of *Pto*DC3000 on *A. thaliana*. Lee et al. also investigated the role of PAA produced by the host during pathogenesis, and observed that *Pto*DC3000 infection perturbs PAA/IAA homeostasis, and that influences the outcome of pathogenesis. Altogether, the work demonstrates the need to understand not only the biosynthesis and role of IAA in plant-microbe interactions, but also other auxins that have not received much attention.

## Conclusions and future challenges

The four studies featured in this Research Topic not only point to the multiple roles of IAA in plant-microbe interactions, but also raise some interesting questions: What proportion of strains from plant-associated or soil microbiomes have the capacity to produce auxin, and in which forms and quantities? Do microbes produce functional auxin conjugates as well or are conjugating enzymes only needed to inactivate excess auxin? Does this variation in auxin production result in different impacts on different types of plant-microbe interactions, such as pathogenesis, symbiosis or general impacts on plant growth and development? Could auxin serve as a signal for microbes, for example as a way to sense the presence of a host, or for communication between microbes and thus shapes microbiome assemblage? We hope that this Research Topic will prompt further investigation into this exciting area of biology.
